# Opsin genes of select treeshrews resolve ancestral character states within Scandentia

**DOI:** 10.1098/rsos.182037

**Published:** 2019-04-24

**Authors:** Gwen Duytschaever, Mareike C. Janiak, Perry S. Ong, Konstans Wells, Nathaniel J. Dominy, Amanda D. Melin

**Affiliations:** 1 Department of Anthropology and Archaeology, University of Calgary, Calgary, Alberta, Canada; 2 Alberta Children's Hospital Research Institute, Calgary, Alberta, Canada; 3 Institute of Biology, University of the Philippines Diliman, Quezon City, Philippines; 4 Department of Biosciences, Swansea University, Wales, UK; 5 Department of Anthropology, Dartmouth College, Hanover, NH, USA; 6 Department of Biological Sciences, Dartmouth College, Hanover, NH, USA; 7 Department of Medical Genetics, University of Calgary, Calgary, Alberta, Canada

**Keywords:** *Dendrogale murina*, northern smooth-tailed treeshrew, *Tupaia everetti*, *Urogale*, Mindanao treeshrew, colour vision

## Abstract

Treeshrews are small, squirrel-like mammals in the order Scandentia, which is nested together with Primates and Dermoptera in the superordinal group Euarchonta. They are often described as living fossils, and researchers have long turned to treeshrews as a model or ecological analogue for ancestral primates. A comparative study of colour vision-encoding genes within Scandentia found a derived amino acid substitution in the long-wavelength sensitive opsin gene (*OPN1LW*) of the Bornean smooth-tailed treeshrew (*Dendrogale melanura*). The opsin, by inference, is red-shifted by *ca* 6 nm with an inferred peak sensitivity of 561 nm. It is tempting to view this trait as a novel visual adaptation; however, the genetic and functional diversity of visual pigments in treeshrews is unresolved outside of Borneo. Here, we report gene sequences from the northern smooth-tailed treeshrew (*Dendrogale murina*) and the Mindanao treeshrew (*Tupaia everetti*, the senior synonym of *Urogale everetti*). We found that the opsin genes are under purifying selection and that *D. murina* shares the same substitution as its congener, a result that distinguishes *Dendrogale* from other treeshrews, including *T. everetti.* We discuss the implications of opsin functional variation in light of limited knowledge about the visual ecology of smooth-tailed treeshrews.

## 1. Introduction

**Figure 1.**
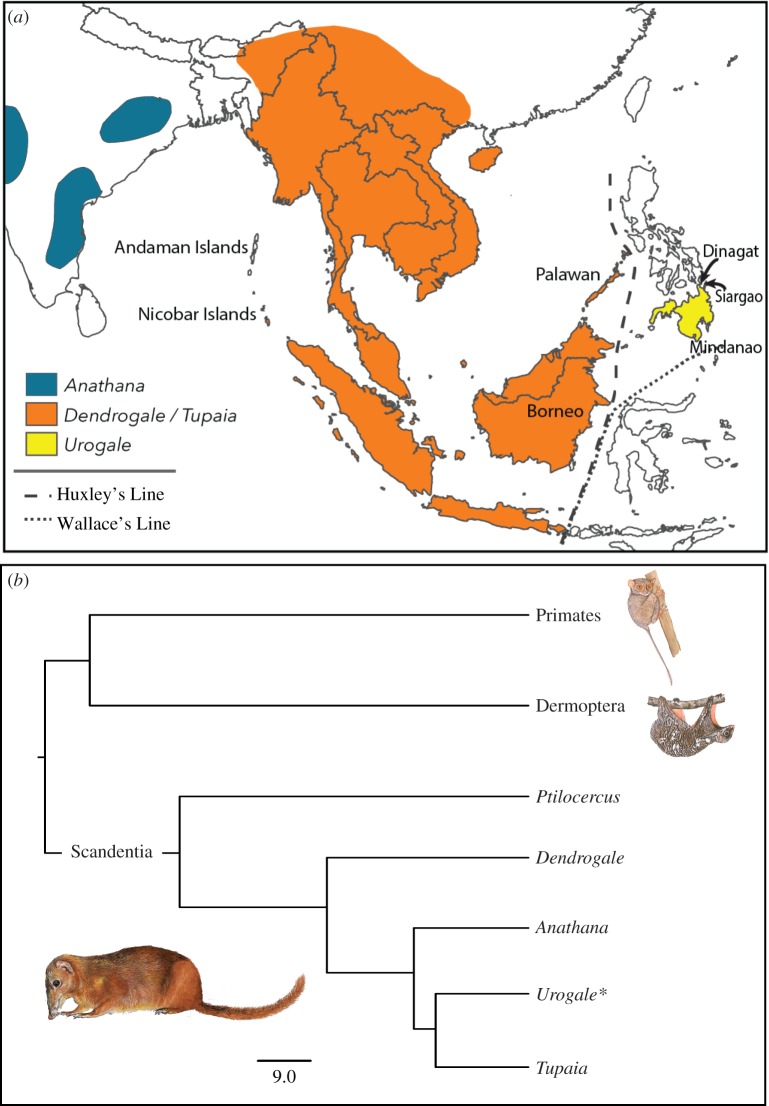
(*a*) Approximate distribution of treeshrew genera in the family Tupaiidae. *Dendrogale* is broadly sympatric with *Tupaia* in northern Borneo and Indochina. Huxley's Line generally corresponds to the edge of the Asian continental shelf and separates oceanic islands from landbridge islands in the Philippines. Redrawn from Roberts *et al*. [1]. (*b*) Phyletic relationships of genera historically recognized in the order Scandentia, which is sister to Dermoptera and Primates (Primatomorpha) in the superordinal group Euarchonta [2,3]. *Recently, *Urogale* has been subsumed into the genus *Tupaia* [2,4,5]. Original artwork by Priscilla Barrett, reproduced with permission.

Treeshrews are small, squirrel-like mammals with a broad distribution from India and southern China through most of Southeast Asia (figure 1*a*). They comprise a single order, Scandentia, in which two families are recognized: Ptilocercidae, containing a nocturnal species, *Ptilocercus lowii,* and Tupaiidae, containing 22 diurnal species [4] in four traditional genera (*Dendrogale*, *Anathana*, *Urogale* and *Tupaia;* figure 1*b*). Scandentia is nested in the superordinal group Euarchonta together with Dermoptera and Primates (figure 1*b*), an affinity that invites the use of treeshrews as a model system for studying a wide range of human disorders, including myopia [6]. Treeshrews are also viewed as ‘living fossils’ [7] and therefore practical models or ecological analogues of ancestral and stem primates [8–14]. Accordingly, Melin *et al*. [15] sequenced the opsin genes of Bornean treeshrews to contextualize the origins of high-acuity colour vision in primates. In *Dendrogale melanura*—the earliest branching tupaiid in their sample—they found an amino acid substitution (_A_180_S_) that translates into a relatively red-shifted long-wavelength sensitive (LWS) opsin. Given that opsins are sensitive to ecological selective pressures [16], it is tempting to interpret this genotype as a novel visual adaptation; however, the full extent of its derivation in Tupaiidae is uncertain. To resolve this uncertainty, data are needed from treeshrew species outside Borneo.

Here we fill two voids by focusing on the only extant congener of *D. melanura*, the northern smooth-tailed treeshrew (*Dendrogale murina*) and the Mindanao treeshrew, a species described as *Tupaia everetti* in 1892 and elevated to a monotypic genus (*Urogale*) in 1905 on the basis of distinguishing morphological traits [17]. This latter nomen prevailed for a century until mounting molecular evidence favoured the subsumption of *Urogale* into *Tupaia* [1,2,18]. *Tupaia everetti* is therefore the senior synonym of *U. everetti*, and re-recognition of *T. everetti* is spreading in the literature [4,5]. Setting this taxonomic reversal aside, *T. everetti* holds interest because it is the earliest branch in crown *Tupaia*, splitting prior to the diversification of *Tupaia* across Southeast Asia (figure 1*b*). It is therefore crucial for pinpointing the origin of differential opsin sensitivities in Tupaiidae.

Accordingly, we examined the sequences of exons known to determine the spectral tuning of the LWS opsin gene (*OPN1LW*) as well as the short-wavelength sensitive opsin gene (*SWS1, OPN1SW*), which has been subject to different selective pressures among taxa within Scandentia and more generally across Euarchonta [15]. We then compared the sequences of *D. murina* and *T. everetti* to those of other treeshrews and primates in order to reconstruct the ancestral character states of opsin genes at two nodes within Tupaiidae.

## 2. Material and methods

### 2.1. Study species and sample collection

**Figure 2.**
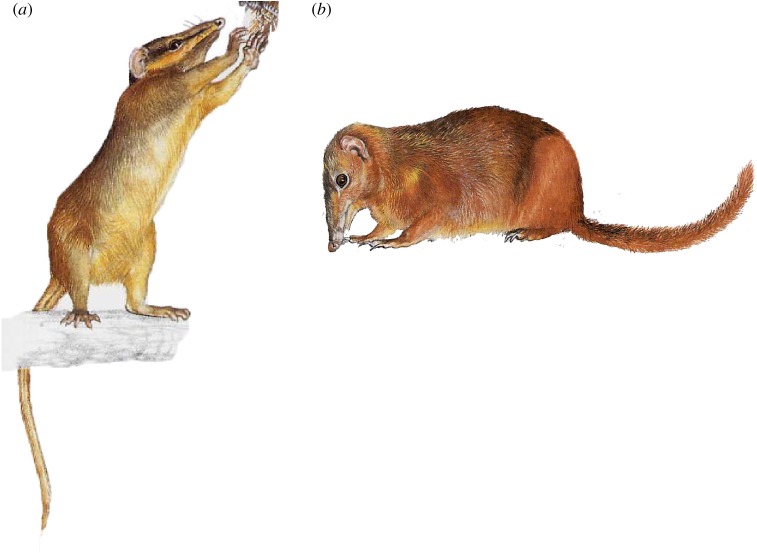
Illustrations of study species: (*a*) the Northern smooth-tailed treeshrew, *Dendrogale murina* and (*b*) the Mindanao treeshrew, *Tupaia everetti*. Original artwork by Priscilla Barrett, reproduced with permission.

*Dendrogale murina* is one of two recognized species in the genus (figure 2*a*). It has a wide distribution throughout Vietnam, Thailand and Cambodia [19] in contrast to its congener (*D. melanura*), which is endemic to montane Borneo [20]. It is also more flexible ecologically, with records ranging from lowland plains to 1500 m across a wide range of habitat conditions, from evergreen forest (at varying stages of degradation), to mixed deciduous forest, to secondary bamboo fields lacking any dicotyledonous canopy, to streamside tangles in rocky savannah [19]. It not only uses the under- and mid-storeys, but also enters the canopy; recent observations come primarily from understorey tangles, especially of bamboo, almost exclusively 30–300 cm above ground level [19]. In Thailand, it has been observed on the branches of fruiting trees [19].

*Tupaia everetti* is the sole scandentian to inhabit the Mindanao Faunal Region, Philippines (figure 2*b*; [17,21]). It has a widespread distribution on Mindanao, preferring montane and mossy forests between 750 and 2250 m a.s.l., but it is also found at lower elevations on neighbouring islands [5]. Limited observations and stomach content analysis indicate a diurnal terrestrial niche and a mixed diet of ground-dwelling insects, other arthropods, fruits and other plant material [22,23]. It is readily differentiated from other tupaiids by its even-haired round tail, elongated snout and large, canine-like second incisors [17]; it is also the largest treeshrew, with captive adults ranging from 270 to 410 g (mean: 314 g; *n* = 7 [24]) and 190 to 342 g (mean: 276 ± 8.0 g; *n* = 10 males; mean: 252 ± 6.3 g; *n* = 18 females [25]). Wild-caught specimens are equally large (range: 235–315 g; *n* = 2 males [26]), but Heaney reported smaller values (mean: 190 g; *n* = 3; cf. Sargis [27]). Data are limited, but specimens of *T. everetti* from Dinagat and Siargao are smaller than those from Mindanao [28], raising the possibility of cryptic speciation on these islands [29].

We extracted DNA from the muscle tissues of museum specimens. The specimen of *D. murina* is accessioned in the University of Alaska Museum (catalogue no. UAM 103000). It was a wild-caught male from the Seima Biodiversity Conservation Area, Mondulkiri, Cambodia. Two specimens of *T. everetti* are housed at the Vertebrate Museum, Institute of Biology, University of the Philippines Diliman, Quezon City (catalogue nos. PNM7496 [adult male] and PNM7497 [juvenile male]). The animals were wild-caught in the Mt Apo Natural Park, Barangay Agco, Kidapawan City, Cotabato, Mindanao. Muscle tissues were biopsied in the field and stored in 99% ethanol.

### 2.2. DNA extraction, amplification and sequencing

Genomic DNA was extracted from muscle tissues using a DNeasy Blood and Tissue Kit (Qiagen) following the manufacturer's instructions. Amino acids at 10 sites on exon 1 of the *OPN1SW* gene determine the spectral tuning of the opsin, with three sites—*86, 90 *and* 93*—primarily governing sensitivity in the violet-blue (400–450 nm) region of the light spectrum [30–32]. The *λ*_max_ of the opsin encoded by *OPN1LW* is determined by five amino acid sites spanning exons 3–5 [33]. Three of these sites are variable in primates (exon 3: 180; exon 5: 277, 285; [34,35]); among treeshrews, genotypes at sites 180, 277 and 285 have recently been reported, with variation between taxa noted at site 180 [15]. We obtained partial opsin sequences for *D. murina* and *T. everetti* by amplifying exons 1, 2–3, 4 and 5 of the *OPN1SW* and exons 3 and 5 of the *OPN1LW*. Although the tuning sites of *OPN1SW* are located on exon 1, variation in gene functionality occurs within Scandentia, and we sequenced additional exons to rule out indels or premature stop codons leading to gene pseudogenization [15].

Polymerase chain reactions (PCRs) were conducted in 25 µl volumes containing 1× KAPA HiFi Readymix (Kapa Biosystems Inc., USA), 1.0 mM each of the forward and reverse primers (electronic supplementary material, table S1) and 200 ng template DNA. Molecular grade water was used as a negative control in all reactions. Thermocycler parameters were as follows: 3 min initial denaturation at 98°C; 35 cycles of 10 s denaturation at 98°C, 30 s annealing at either 58°C (for *OPN1SW* exons 1 and 4) or 60°C (for *OPN1SW* exons 2–3 and 5 and *OPN1LW* exons 3 and 5) and 30 s extension at 72°C, followed by a 5 min final extension at 72°C.

Amplification of target sequences was confirmed on a 1.5% agarose gel in 1 X Tris Borate EDTA (TBE) buffer. PCR products were purified using EXOsap-IT (Affymetrix, USA) following the manufacturer's protocol. If non-target sequences were also amplified, target amplicons were excised and purified using the Purelink Gel Extraction kit (Life Technologies Inc.). Purified PCR products were directly Sanger sequenced on sense and antisense strands at the University of Calgary Core DNA Sequencing Facility (Faculty of Medicine, University of Calgary, Alberta, Canada) using an Applied Biosystems 3730×l 96 capillary DNA Analyzer.

### 2.3. Data analysis

We assembled and edited *OPN1SW* and *OPN1LW* sequences in Geneious v. 10.0.3 (Biomatters) using the Clustal W function with manual refinement. We aligned them to opsin sequences from *Homo sapiens* and published treeshrew species: *Ptilocercus lowii, Dendrogale melanura,* and six species of *Tupaia* (*T. minor, T. belangeri, T. tana, T. montana, T. longipes and T. gracilis*; electronic supplementary material, table S2). We translated the coding regions into amino acid sequences, which we used to infer colour vision phenotype. Site numbers in our alignments correspond to the position of the amino acid in the human SWS1 and M/LWS pigments. We used the Protein Variation Effect Analyzer (http://provean.jcvi.org/; PROVEAN) tool to predict the functional implications of other nonsynonymous mutations [36,37]. PROVEAN predicts deleterious polymorphisms, but the functional effect of nonsynonymous mutations that change the amino acid will be predicted neutral when the properties of the amino acid do not change drastically (e.g. due to hydrophobicity). We tested for evidence of purifying or positive selection in *OPN1SW* and *OPN1LW* using codeml free-ratio branch models implemented in phylogenetic analysis by maximum likelihood (PAML) [38]. Outgroups for PAML analyses were two primates with functional opsin genes, *Alouatta palliata* (*OPN1SW*: AH005790.1; *OPN1LW*: AB809459.1) and *Tarsius bancanus* (*OPN1SW*: AB111463.1 and *OPN1MW*: AB675927.1) (electronic supplementary material, table S2). We also used codeml site models to test whether any sites, including the tuning sites are under positive selection. We did not include *P. lowii* in the PAML analysis of the *OPN1SW* gene due to low sequence coverage. This nocturnal species has a predicted *OPN1SW* pseudogene of ancient origin and relaxed selection pressures have previously been reported [15].

## 3. Results

We successfully sequenced partial exons 1–5 of *OPN1SW,* and exons 3 and 5 of *OPN1LW* for the *D. murina* and *T. everetti* specimens (electronic supplementary material, figure S1). Among scandentian sequences, the mean amino acid divergence (mean amino acid difference per sequence/total amino acids analysed, s.e. over 1000 bootstrap replicates) for *OPN1SW* was 1.02% divergence (3.524/347, s.e. = 0.822). The amino acid divergence for *OPN1LW* partial sequences was 0.96% divergence (1.311/136, s.e. = 0.567).

### 3.1. *OPN1SW* evolution

**Figure 3.**
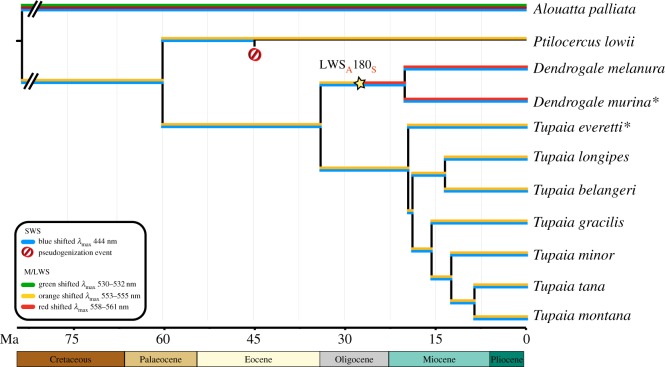
Phyletic relationships and divergence dates for primates and treeshrews were based on TimeTree [39] (accessed February 2019) and published estimates [2]. Branch colours correspond with the function and spectral tuning of opsin photopigments. Pseudogenization events are marked with a diagonally bisected circle. The inferred long-wave shift from orange to red sensitivity in the LWS opsin of the genus *Dendrogale* is marked with a star, along with the amino acid substitution responsible. The timing of this shift in sensitivity is unknown. Species sequenced in this study are indicated with an asterisk.

Relative to *T. everetti* and other *Tupaia* species*, D. murina* has four derived nonsynonymous variants causing amino acid differences: _G_111_A_ and _V_119_C_ in exon 1, _T_159_A_ in exon 2, and _S_224_T_ in exon 3 (electronic supplementary material, figure S1). The first two mutations are shared with *D. melanura*, but missing sequence data for *D. melanura* prevent assessing whether the last two are shared or unique to *D. murina*. At site 322, the *OPN1SW* amino acid sequence of *D. murina* has retained the ancestral state Cys322, relative to its congener, which has a derived nonsynonymous variant Phe322. All these nonsynonymous mutations are predicted to be neutral using PROVEAN, scores = −1.484 (site 111), 2.346 (site 119), −0.341 (site 159), 1.773 (site 224) and −1.903 (site 322); cut-off = −2.5)*. Tupaia everetti* differs from *Tupaia* species in possessing L rather than Q at site 28. This may be the ancestral condition for Tupaiidae and possibly for Scandentia, as it is shared with *D. melanura*. Data at these sites from *P. lowii* would help to further resolve this but are presently unavailable. There are two derived substitutions unique to *T. everetti*: exon 1, _A_120_T_, exon 5, _R_323_K_ (electronic supplementary material, figure S1). All mutations were predicted to be neutral (PROVEAN scores = 4.742 (site 28), 0.977 (site 120) and −0.299 (site 323); cut-off = −2.5). Interestingly, a derived mutation in *T. longipes*, _F_115_V,_ may be deleterious (PROVEAN score, −5.951). Overall, based on broad conservation of amino acids, including the main spectral tuning sites Tyr86, Ser90 and Val93 (exon 1), the *λ*_max_ of the SWS1 opsin protein of both *D. murina* and *T. everetti* is predicted to be around 444 nm [15,30,32] (figure 3).

### 3.2. *OPN1LW* evolution

Two derived nonsynonymous mutations (_A_180_S_ and _V_182_I_, exon 3) are present in the *OPN1LW* gene sequence of *D. murina *and* D. melanura* compared to other treeshrews, indicating a shared origin in the last common ancestor of extant *Dendrogale* species (electronic supplementary material, figure S1). Nonsynonymous substitutions at site 180 and 182 were predicted to be neutral (PROVEAN respective scores = −0.302 and 0.133; cut-off = −2.5). However, site 180 is a known opsin tuning site (figure 3). We did not detect any derived nonsynonymous mutations in the *OPN1LW* sequence of *T. everetti*. Based on the three-site composition of SYT at sites 180, 277 and 285, *D. melanura* and *D. murina* are predicted to be long-wavelength shifted, and have a *λ*_max_ value of *ca* 561 nm, as opposed to shorter *λ*_max_ values for other treeshrews. We predict that the *T. everetti* LWS opsin protein (three-site composition of AYT) has a similar *λ*_max_ to *Tupaia* and *Ptilocercus*, *ca* 555 nm.

### 3.3. Evaluation of selective pressures

The codeml branch models for *OPN1SW* indicated purifying selection acting on all species (*ω* < 0.312), but supported a model of branch-specific differences in the strength of selection (*p* = 0.045, *Likelihood Ratio* (*LR*) = 29.25), showing stronger purifying selection acting on *D. murina* (*ω* = 0.0001) than on *T. everetti* (*ω* = 0.312). The codeml branch model did not find species-specific differences in the strength of selection acting on *OPN1LW*, indicating that the gene is under purifying selection (*ω* = 0.165) in all examined species, including *D. murina* and *T. everetti*. We did not find evidence of positively selected sites in our codeml site models for either *OPN1SW* (*p* = 1, *LR* = 0) or *OPN1LW* (*p* = 0.228, *LR* = 2.957).

## 4. Discussion

We found that the short-wavelength sensitive opsin gene (*OPN1SW*) is intact in both *D. murina* and *T. everetti*. The amino acid composition of 10 spectral tuning sites, including the three most influential (Tyr86, Ser90, Val93) predicts a *λ*_max_ of 444 nm, an inferred phenotype that unites *D. murina* and *T. everetti* together with every tupaiid examined to date [15]. In addition, we found that the long-wavelength sensitive opsin gene (*OPN1LW*) is functionally variable in the two study species. Despite PROVEAN's neutral prediction, *in vitro* site-directed mutagenesis of the LWS pigment has demonstrated that the single site mutation _A_180_S_ significantly shifts the *λ*_max_ of the pigment [33]. Notably, *D. murina* shares the _A_180_S_ substitution with its congener *D. melanura* [15]. The LWS opsins of *Dendrogale* are therefore predicted to have a red-shifted *λ*_max_ of 561 nm, whereas those of all other scandentians, including *T. everetti* (present results), are predicted to have a *λ*_max_ of 555 nm.

Our results suggest that the colour vision of *T. everetti* is practically identical to that of other *Tupaia* species despite its relatively large body size and restricted distribution. This result is consistent with recent shared ancestry [2], and it suggests comparable visual ecologies. Indeed, observations of habitat and resource use by these species speak to similar preferences for insects and fruit in the forest understorey [19,22,40,41]. It is possible that the 444/555 nm opsin combination is a ‘multi-purpose’ dichromacy with functionality across a wide range of understorey light conditions. Given the strict conservation of opsin genes in *Tupaia*, including the spectral tuning sites, it is rather surprising that *Dendrogale* is predicted to express a red-shifted LWS opsin, which suggests unique selective pressures on the colour vision of this lineage. The implications of this 6 nm red-shift are discussed below.

The split between Ptilocercidae and Tupaiidae occurred *ca* 60 Ma, preceding the emergence of *Dendrogale*, the earliest branching tupaiid, *ca* 35 Ma; divergence of the two extant congeners, *D. melanura* and *D. murina*, is estimated at *ca* 21 Ma [2]. Our results raise the possibility that the common ancestor of *Dendrogale* occupied a distinct visual niche that favoured the fixation of a red-shifted opsin, once this novel mutation arose. One possibility is that *Dendrogale* is adapted to relatively open habitats with greater exposure to unfiltered daylight. Such light is relatively enriched in longer wavelengths compared to foliage-filtered downwelling light [42,43]. In consequence, redder objects should appear brighter in open habitats illuminated with redder light; however, the nature of these putative objects (foods, russet-coloured predators) is uncertain.

Some support for this ‘open habitat’ hypothesis is evident in the natural history of *D. murina*—it is found in degraded evergreen forest, mixed deciduous forest, bamboo fields without a dicotyledonous canopy, as well as streamside tangles in rocky savannahs [19]. It is also evident in the differences between two sympatric montane species in Mount Kinabalu National Park, Sabah, Borneo [44]. Smooth-tailed treeshrews (*D. melanura*) are found around elevations of well above 900 m, the point at which another species of montane treeshrew, *Tupaia montana*, replaces its lowland congeners *T. gracilis*, *T. longipes*, *T. minor* and *T. tana* [40,44]. The elevational overlap of *D. melanura* and *T. montana* is telling, as the smaller *D. melanura* has a limited range, patchier distribution, and prefers relatively open areas around mossy boulders, whereas *T. montana* prefers greater forest cover and uses a larger range of montane habitats, as reflected by its higher abundance in trap records ([2,40]; K. Wells 2009–2010, personal observation). Importantly, our finding indicates deep antiquity for the red-shifted *OPN1LW* in *Dendrogale*. This interpretation argues against the possibility of recent character displacement; i.e. that the colour vision of *D. melanura* is the result of competition with *T. montana* and niche-divergence in diet, microhabitat or other aspects of visual ecology [15]. However, early opsin divergence between *Dendrogale* and other tupaiids may have facilitated current sympatry via different microhabitat preferences, i.e. habitat patches with less intensive canopy cover versus greener, denser canopy cover, a hypothesis that invites testing through behavioural observation and characterization of downwelling light. Overall, there is a need to better understand fine-scale habitat use by sympatric species at different times of the year and to characterize the light conditions in different microhabitats.

Finally, it is alternatively possible that the _A_180_S_ substitution in *Dendrogale* did not impact the fitness of individuals, but spread through an ancestral population neutrally. If so, this may point to a bottleneck event in the history of this genus, as neutral mutations are unlikely to become fixed in large populations [45]. Future examination of the population genomics of extant *Dendrogale* species and estimations of N_E_ and past population contractions and expansions may allow us to favour or rule out this hypothesis. Additionally, increased sampling of *OPN1LW* in these species will also allow us to rule out *OPN1LW* polymorphisms, which are common in neotropical primates, but unknown outside of the primate order [15,35,46].

## 5. Conclusion

We present new data on the opsin gene sequences of the northern smooth-tailed treeshrew, *Dendrogale murina*, and the Mindanao treeshrew, *Tupaia everetti*. The gene codings for both short-wavelength sensitive and long-wavelength sensitive opsins are under purifying selection in each species and presumed to be functional. *Dendrogale murina* shared a derived amino acid with its congener, *D. melanura*, at site 180 of the *OPN1LW*, which should cause a shift in the sensitivity towards reddish light. This may indicate the common ancestor of extant *Dendrogale* taxa occupied a relatively more open habitat—richer in light unfiltered by green foliage—and may contribute to present-day niche separation among sympatric diurnal treeshrews. We end by noting that lack of availability of DNA from *Anathana* precluded analysis of the opsins of the remaining genus in the order Scandentia, but in the future this would shed additional light on pressures shaping treeshrew visual ecology.

## Supplementary Material

Electronic Supplementary Material

Reviewer comments

## Data accessibility

Nucleotide sequences were deposited in GenBank (accession nos. MH129025, KY825132 and KY825133 (*OPN1SW*), and MH129024, KY825134 and KY825135 (*OPN1LW*)). *OPN1SW* and *OPN1LW* nucleotide and amino acid sequence alignments are available in electronic supplementary material, figure S1 and at the Dryad Digital Repository (https://doi.org/10.5061/dryad.56b1t40) [48].

## References

[1] Roberts TE, Sargis EJ, Olson LE 2009 Networks, trees, and treeshrews: assessing support and identifying conflict with multiple loci and a problematic root. Syst. Biol. 58, 257–270. (10.1093/sysbio/syp025) 20525582 PMC2715937

[2] Roberts TE, Lanier HC, Sargis EJ, Olson LE 2011 Molecular phylogeny of treeshrews (Mammalia: Scandentia) and the timescale of diversification in Southeast Asia. Mol. Phylogenet. Evol. 60, 358–372. (10.1016/j.ympev.2011.04.021) 21565274

[3] Mason VC et al*.* 2016 Genomic analysis reveals hidden biodiversity within colugos, the sister group to primates. Sci. Adv. 2, e1600633 (10.1126/sciadv.1600633) 27532052 PMC4980104

[4] Hawkins MT 2018 Family Tupaiidae (treeshrews). In Handbook of the mammals of the world, volume 8 insectivores, sloths and colugos (eds Wilson DE, Mittermeier RA, Francois TL), pp. 242–269. Barcelona, Spain: Lynx Edicions.

[5] Tabaranza B, Gonzalez JC, Rosell-Ambal RGB, Heaney L 2017 *Tupaia everetti* (amended version of 2016 assessment). *IUCN Red List of Threatened Species*. See https://www.iucnredlist.org/species/22784/114120582 (accessed on 28 February 2019).

[6] Fan Y et al*.* 2013 Genome of the Chinese tree shrew. Nat. Commun. 4, 1426 (10.1038/ncomms2416) 23385571

[7] Li Q, Ni X 2016 An early oligocene fossil demonstrates treeshrews are slowly evolving ‘living fossils’. Sci. Rep. 6, 18627 (10.1038/srep18627) 26766238 PMC4725336

[8] Simpson GG 1965 Long-abandoned views. Science 147, 1397 (10.1126/science.147.3664.1397) 17776614

[9] Jenkins FA 1974 Tree shrew locomotion and the origins of primate arborealism. In Primate locomotion (ed. Jenkins FA), pp. 85–115. New York, NY: Academic Press.

[10] Tattersall I 1984 The tree-shrew, *Tupaia*: a ‘living model’ of the ancestral primate? In Living fossils (eds Eldredge N, Stanley SM), pp. 32–37. New York, NY: Springer.

[11] Gebo DL 2004 A shrew-sized origin for primates. Am. J. Phys. Anthropol. 47, 40–62. (10.1002/ajpa.20154) 15605387

[12] Sargis EJ 2004 New views on tree shrews: the role of tupaiids in primate supraordinal relationships. Evol. Anthropol. 13, 56–66. (10.1002/evan.10131)

[13] Melin AD et al*.* 2014 Calcium and carbon stable isotope ratios as paleodietary indicators. Am. J. Phys. Anthropol. 154, 633–643. (10.1002/ajpa.22530) 24839035

[14] Silcox MT, Sargis EJ, Bloch JI, Boyer DM 2015 Primate origins and supraordinal relationships: morphological evidence. In Handbook of paleoanthropology (eds Henke W, Tattersall I), pp. 1053–1081. Berlin, Germany: Springer.

[15] Melin AD et al*.* 2016 Euarchontan opsin variation brings new focus to primate origins. Mol. Biol. Evol. 33, 1029–1041. (10.1093/molbev/msv346) 26739880 PMC4776711

[16] Davies WIL, Collin SP, Hunt DM 2012 Molecular ecology and adaptation of visual photopigments in craniates. Mol. Ecol. 21, 3121–3158. (10.1111/j.1365-294X.2012.05617.x) 22650357

[17] Lyon MW 1913 Treeshrews: an account of the mammalian family tupaiidae. Washington, DC: US Gov't Print Off.

[18] Olson LE, Sargis EJ, Martin RD 2004 Phylogenetic relationships among treeshrews (Scandentia): a review and critique of the morphological evidence. J. Mamm. Evol. 11, 49–71. (10.1023/B:JOMM.0000029145.28207.6d)

[19] Timmins RJ, Duckworth JW, Robson CR, Walston JL 2003 Distribution, status and ecology of the mainland slender-tailed treeshrew *Dendrogale murina*. Mamm. Rev. 33, 272–283. (10.1046/j.1365-2907.2003.00013.x)

[20] Corbet GB, Hill JE 1992 The mammals of the Indomalayan region: a systematic review. Oxford, UK: Oxford University Press.

[21] Heaney LR et al*.* 1998 A synopsis of the mammalian fauna of the Philippine Islands. Fieldiana. Zool. 88, 1–61. (10.5962/bhl.title.3419)

[22] Wharton CH et al*.* 1950 Notes on the Philippine tree shrew *Urogale everetti* Thomas. J. Mammal. 31, 352–354. (10.1093/jmammal/31.3.352)

[23] Rabb GB, Getty RE, Williamson WM, Lombard LS 1966 Spontaneous diabetes mellitus in tree shrews, *Urogale everetti*. Diabetes 15, 327–330. (10.2337/diab.15.5.327) 5327707

[24] Fooden J 1961 Urinary amino acids of non-human primates. Zoologica 46, 167–180.

[25] Nelson LE, Asling CW 1962 Metabolic rate of tree-shrews (*Urogale everetti*). Proc. Soc. Exp. Biol. Med. 109, 602–604. (10.3181/00379727-109-27281) 14478924

[26] Bauchot R, Stephan H 1966 Données nouvelles sur l'encéphalisation des insectivores et des prosimiens. Mammalia 30, 160–196. (10.1515/mamm.1966.30.1.160)

[27] Sargis EJ 2000 The functional morphology of the postcranium of Ptilocercus and tupaiines (Scandentia, Tupaiidae): implications for the relationships of primates and other Archontan mammals. PhD, City University of New York.

[28] Heaney LR, Rabor DS. (1982).

[29] Brown RM et al*.* 2014 Conservation genetics of the Philippine tarsier: cryptic genetic variation restructures conservation priorities for an island archipelago primate. PLoS ONE 9, e104340 (10.1371/journal.pone.0104340) 25136854 PMC4138104

[30] Yokoyama S, Starmer WT, Takahashi Y, Tada T 2006 Tertiary structure and spectral tuning of UV and violet pigments in vertebrates. Gene 365, 95–103. (10.1016/j.gene.2005.09.028) 16343816 PMC2810422

[31] Hunt DM, Carvalho LS, Cowing JA, Parry JWL, Wilkie SE, Davies WL, Bowmaker JK 2007 Spectral tuning of shortwave-sensitive visual pigments in vertebrates. Photochem. Photobiol. 83, 303–310. (10.1562/2006-06-27-IR-952) 17576346

[32] Carvalho LS, Davies WL, Robinson PR, Hunt DM 2012 Spectral tuning and evolution of primate short-wavelength-sensitive visual pigments. Proc. R. Soc. B 279, 387–393. (10.1098/rspb.2011.0782) PMC3223675 21697177

[33] Yokoyama S, Yang H, Starmer WT 2008 Molecular basis of spectral tuning in the red- and green-sensitive (M/LWS) pigments in vertebrates. Genetics 179, 2037–2043. (10.1534/genetics.108.090449) 18660543 PMC2516078

[34] Kawamura S 2016 Color vision diversity and significance in primates inferred from genetic and field studies. Genes Genomics 38, 779–791. (10.1007/s13258-016-0448-9) 27594978 PMC4987397

[35] Jacobs RL et al*.* 2017 Novel opsin gene variation in large-bodied, diurnal lemurs. Biol. Lett. 13, 20170050 (10.1098/rsbl.2017.0050) 28275167 PMC5377041

[36] Choi Y 2012 A fast computation of pairwise sequence alignment scores between a protein and a set of single-locus variants of another protein. In Proc. of the ACM Conference on Bioinformatics, Computational Biology and Biomedicine, Orlando, FL, 7–10 October, pp. 414–417. New York, NY: ACM.

[37] Choi Y, Sims GE, Murphy S, Miller JR, Chan AP 2012 Predicting the functional effect of amino acid substitutions and indels. PLoS ONE 7, e46688 (10.1371/journal.pone.0046688) 23056405 PMC3466303

[38] Yang Z 2007 PAML 4: phylogenetic analysis by maximum likelihood. Mol. Biol. Evol. 24, 1586–1591. (10.1093/molbev/msm088) 17483113

[39] Hedges SB, Dudley J, Kumar S 2006 TimeTree: a public knowledge-base of divergence times among organisms. Bioinformatics 22, 2971–2972. (10.1093/bioinformatics/btl505) 17021158

[40] Emmons LH 2000 Tupai: a field study of Bornean treeshrews. Berkeley, CA: University of California Press.

[41] Wells K, Pfeiffer M, Lakim MB, Kalko EKV 2006 Movement trajectories and habitat partitioning of small mammals in logged and unlogged rain forests on Borneo. J. Anim. Ecol. 75, 1212–1223. (10.1111/j.1365-2656.2006.01144.x) 16922857

[42] Endler JA 1993 The color of light in forests and its implications. Ecol. Monogr. 63, 1–27. (10.2307/2937121)

[43] Moritz GL, Ong PS, Perry GH, Dominy NJ 2017 Functional preservation and variation in the cone opsin genes of nocturnal tarsiers. Phil. Trans. R. Soc. B 372, 20160075 (10.1098/rstb.2016.0075) 28193820 PMC5312025

[44] Payne J, Francis CM 2007 A field guide to the mammals of Borneo (2007). *Revised edition*. Kota Kinabalu, Sabah, Malaysia: The Sabah Society.

[45] Hartl DL, Clark AG 2006 Principles of population genetics, 4th edn. Sunderland, MA: Sinauer Associates, Inc.

[46] Jacobs GH 2009 Evolution of colour vision in mammals. Phil. Trans. R. Soc. B 364, 2957–2967. (10.1098/rstb.2009.0039) 19720656 PMC2781854

[47] Sikes RS et al 2011 Guidelines of the American Society of Mammalogists for the use of wild mammals in research. J. Mammal. 92, 235–253. (10.1644/10-MAMM-F-355.1) PMC5909806 29692469

[48] Duytschaever G, Janiak MC, Ong PS, Wells K, Dominy NJ, Melin AD 2019 Opsin genes of select treeshrews resolve ancestral character states within Scandentia Dryad Digital Repository. (10.5061/dryad.56b1t40) PMC6502361 31183134

