# Supplementary material for: Opsin genes of select treeshrews resolve ancestral character states within Scandentia
Source: R Soc Open Sci. 2019 Apr 24;6(4):182037. doi: 10.1098/rsos.182037 (PMC6502361; doi:10.1098/rsos.182037)
Supplement: Electronic Supplementary Material [file rsos182037supp1.pdf]

Royal Society Open Science

Electronic Supplementary Material for:

Opsin genes of select treeshrews resolve ancestral character states within Scandentia

Gwen Duytschaever[1], Mareike C. Janiak[1], Perry S. Ong[2], Konstans Wells[3], Nathaniel J. Dominy[4,5], Amanda D. Melin[1,6,7*]

[1]Department of Anthropology & Archaeology, University of Calgary, Calgary, AB, Canada. [2]Institute of Biology, University of the Philippines Diliman, Quezon City, Philippines. [3]Department of Biosciences, Swansea University, Wales, UK. [4]Department of Anthropology, Dartmouth College, Hanover, NH, USA,[5]Department of Biological Sciences, Dartmouth College, Hanover, NH, USA. [6]Department of Medical Genetics, University of Calgary, Calgary, AB, Canada; [7]Alberta Children's Hospital Research Institute, Calgary, AB, Canada. [*]e-mail: amanda.melin@ucalgary.ca

This PDF file includes:

Supplementary Tables S1 and S2

Supplementary Figure S1

Table S1 Primers and annealing temperatures used in polymerase chain reactions to amplify partial *OPN1SW* and *OPN1LW* opsin genes in *Tupaia everetti* and *Dendrogale murina*

Gene	Region	Forward (5' to 3')	Reverse (5' to 3')	T (°C)
OPN1SW	Exon 1	GTA CCA CCT TGC CCC TGT CT	CCT TTC CCC TGC AGT ACC T	58
	Exon 2-3	GGT GAT AGG CTG GTC ATT GG	CCC AGC AGC TGA GAG TAG GA	60
	Exon 4	GCT CAG CAG CAG GAG TCA G	TTC ATG AAG CAG TAG ATG ATG G	58
	Exon 5	ATG AGG CGT CTT TTC CAC AC	TGG CTT TGT TAG CAG GAA GG	60
OPN1LW	Exon 3	CAT CAC GGG GCT CTG GTC	CTG CTC CAA CCA AAG ATG G	60
	Exon 5	AGG CTG AGA AGG AGG TGA CA	GTG GCA CTT TTG GCG AAG TA	60

Table S2 Accession numbers for *OPN1SW* and *OPN1LW* opsin gene sequences used as references, and for species newly sequenced in this study

**Species**	***OPN1SW***	***OPN1LW***	**Source**
Homo sapiens	NM020061.5	NM020061.5	Genbank
Alouatta palliata	AH005790.1	AB809459.1	[11]
Tarsius bancanus	AB111463.1	AB675927.1	[11]
Tupaia belangeri	ALAR00000000.1	ALAR00000000.1	Genbank
Tupaia gracilis	KU255121	KU255132	[11]
Tupaia longipes	KU255124	KU255131	[11]
Tupaia minor	KU255122	KU255133	[11]
Tupaia montana	KU255125	KU255135	[11]
Tupaia tana	KU255123	KU255134	[11]
Ptilocercus lowii	KU255127	KU255129	[11]
Dendrogale melanura	KU255126	KU255130	[11]
***Tupaia everetti* DMP298**	KY825132	KY825134	Current study
***Tupaia everetti* DMP322**	KY825133	KY825135	Current study
***Dendrogale murina* UAM 103000**	MH129025	MH129024	Current study

Figure S1. Nucleotide and amino acid sequences of a) *OPN1SW* (exons 1, 2, 3, 4 and 5) and b) *OPN1LW* (exons 3 and 5) of Scandentia species included in the analyses aligned against opsin sequences from *Homo sapiens*. The key spectral tuning sites (*OPN1SW* 46, 49, 52, 86, 90, 93, 97, 114, 116 and 118; *OPN1LW* 180, 277, 285 and 308) are indicated with red boxes. Site numbers correspond to the position of the amino acid in the human SWS1 and M/LWS pigments. The sequences highlighted in light grey were sequenced in this study. Missing sequence data are indicated with a dashed line.

a) *OPN1SW*

Nucleotide sequences

OPN1SW	**Exon 1**																			
			5					10					15					20		
Homo sapiens	ATG	AGA	AAA	ATG	TCG	GAG	GAA	GAG	---	TTT	TAT	CTG	TTC	AAA	AAT	ATC	TCT	TCA	GTG	GGG
Ptilocercus lowii	---	---	---	---	---	---	---	---	---	---	---	---	---	---	---	---	---	---	---	---
Dendrogale melanura	---	---	---	---	---	---	---	---	---	---	---	---	---	---	---	---	---	---	---	---
Dendrogale murina	---	---	---	---	---	---	---	---	---	---	---	---	---	---	---	---	---	---	---	---
Tupaia everetti	---	---	---	---	---	---	---	---	---	---	---	---	---	---	---	---	---	---	---	---
Tupaia minor	..A	..C	..G	...	..A	..A	...	...	GAG	...	...	...	...	..G	..C	GC.	..C	.TG	...	...
Tupaia belangeri	..A	..C	..G	...	..A	..A	...	...	GAG	...	...	...	...	..G	..C	GC.	..C	.TG	...	...
Tupaia tana	..A	..C	..G	...	..A	..A	...	...	GAG	...	...	...	...	..G	..C	GC.	..C	.TG	...	...
Tupaia longipes	..A	..C	..G	...	..A	..A	...	...	GAG	...	...	...	...	..G	..C	GC.	..C	.TG	...	...
Tupaia montana	..A	..C	..G	...	..A	..A	...	...	GAG	...	...	...	...	..G	..C	GC.	..C	.TG	...	...
Tupaia gracilis	..A	..C	..G	...	..A	..A	...	...	GAG	...	...	...	...	..G	..C	GC.	..C	.TG	...	...

OPN1SW **Exon 1**

			25					30					35					40		
Homo sapiens	CCG	TGG	GAT	GGG	CCT	CAG	TAC	CAC	ATT	GCC	CCT	GTC	TGG	GCC	TTC	TAC	CTC	CAG	GCA	GCT
Ptilocercus lowii	---	---	---	---	---	---	---	---	---	---	---	---	---	---	---	---	---	---	---	---
Dendrogale melanura	---	---	---	---	---	TT.	...	...	C..	...	...	...	...	...	...	C..	...	...	...	..C
Dendrogale murina	---	---	---	---	---	--.	...	...	C..	...	...	...	...	...	...	C..	..G	...	...	..C
Tuapaia everetti	---	---	---	---	---	TT.	...	...	C..	...	...	...	...	...	..T	C.T	...	...	...	..C
Tupaia minor	...	...	...	..A	...	...	...	...	C..	...	...	...	...	...	..T	C.T	...	...	...	..C
Tupaia belangeri	...	...	...	..A	...	...	...	...	C..	...	...	...	...	...	..T	C.T	...	...	...	..C
Tupaia tana	...	...	...	..A	...	...	...	...	C..	...	...	...	...	...	..T	C.T	...	...	...	..C
Tupaia longipes	...	...	...	..A	...	..A	...	...	C..	...	...	...	...	...	..T	C.T	...	...	...	..C
Tupaia montana	...	...	...	..A	...	...	...	...	C..	...	...	...	...	...	..T	C.T	...	...	...	..C
Tupaia gracilis	...	...	...	..A	...	...	...	...	C..	...	...	...	...	...	..T	C.T	...	...	...	..C

OPN1SW **Exon 1**

			45	46			49	50		52			55					60		
Homo sapiens	TTC	ATG	GGC	**ACT**	GTC	TTC	**CTT**	ATA	GGG	**TTC**	CCA	CTC	AAT	GCC	ATG	GTG	CTG	GTG	GCC	ACA
Ptilocercus lowii	---	---	---	---	---	---	---	---	---	---	---	---	---	---	---	---	---	---	---	---
Dendrogale melanura	...	...	...	TT.	...	...	T..	G..	...	ACA	...	...	...	...	TCA	...	...	...	...	...
Dendrogale murina	...	...	...	TT.	...	...	T..	G..	...	ACA	...	...	...	...	TCA	...	...	...	...	...
Tupaia everetti	...	...	...	TT.	...	...	T..	G..	...	ACA	..G	...	...	...	TCA	...	...	...	...	..G
Tupaia minor	...	...	...	TT.	...	...	T..	G..	...	ACA	..G	...	...	...	TCA	...	...	...	...	..G
Tupaia belangeri	...	...	...	TT.	...	...	T..	G..	...	ACA	..G	...	...	...	TCA	...	...	...	...	..G
Tupaia tana	...	...	...	TT.	...	...	T.C	G..	...	ACA	..G	...	...	...	TCA	...	...	...	...	..G
Tupaia longipes	...	...	...	TT.	...	...	T..	G..	...	ACA	..G	...	...	...	TCA	...	...	...	...	..R
Tupaia montana	...	...	...	TT.	...	...	T.C	G..	...	ACA	..G	...	...	...	TCA	...	...	...	...	...
Tupaia gracilis	...	...	...	TT.	...	...	T.C	G..	...	ACA	..G	...	...	...	TCA	...	...	...	...	..G

OPN1SW **Exon 1**

			65					70					75					80		
Homo sapiens	CTG	CGC	TAC	AAA	AAG	TTG	CGG	CAG	CCC	CTC	AAC	TAC	ATT	CTG	GTC	AAC	GTG	TCC	TTC	GGA
Ptilocercus lowii	---	---	---	---	---	---	---	---	---	---	---	---	---	---	---	---	---	---	---	---
Dendrogale melanura	A..	...	...	.G.	...	...	..T	...	..A	...	...	...	..C	...	...	...	A.A	...	..T	..G
Dendrogale murina	A..	...	...	.G.	...	...	..T	...	..A	...	...	...	..C	...	...	...	A.A	...	..T	..G
Tupaia everetti	A..	...	...	.G.	...	...	..C	...	...	..T	...	...	..C	...	...	...	A.A	...	..T	..G
Tupaia minor	A..	...	...	.G.	...	...	..C	...	...	..T	...	...	..C	...	...	...	A.A	...	..T	..G
Tupaia belangeri	A..	...	...	.G.	...	...	..C	...	...	..T	...	...	..C	...	...	...	A.A	...	..T	..G
Tupaia tana	A..	...	...	.G.	...	...	..C	...	...	..T	...	...	..C	...	...	...	A.A	...	..T	..G
Tupaia longipes	A..	...	...	.G.	...	...	..C	...	...	..T	...	...	..C	...	...	...	A.A	...	..T	..G
Tupaia montana	A..	...	...	.G.	...	...	..C	...	...	..T	...	...	..C	...	...	...	A.A	...	..T	..G
Tupaia gracilis	A..	...	...	.G.	...	...	..C	...	...	..T	...	...	..C	...	...	...	A.A	...	..T	..G

OPN1SW **Exon 1**

			85	**86**				90 **90**			**93**		95		**97**			100		
Homo sapiens	GGC	TTC	CTC	**CTC**	TGC	ATC	TTC	**TCT**	GTC	TTC	**CCT**	GTC	TTC	GTC	**GCC**	AGC	TGT	AAC	GGA	TAC
Ptilocercus lowii	---	---	---	---	---	---	---	---	---	---	---	---	---	---	---	---	---	---	---	---
Dendrogale melanura	..T	...	...	TA.	...	...	...	...	...	...	GTG	...	...	C.T	AA.	...	...	C.T	...	...
Dendrogale murina	..T	...	...	TA.	...	...	...	...	...	...	GTG	...	...	C.T	AA.	...	...	C.T	...	...
Tupaia everetti	...	...	...	TA.	...	...	...	...	...	...	GT.	...	...	C.T	AA.	...	..C	C.T	...	...
Tupaia minor	...	...	...	TA.	...	...	...	...	...	...	GT.	...	...	C.T	AA.	...	..C	C.T	...	...
Tupaia belangeri	...	...	...	TA.	...	...	...	...	...	...	GT.	...	...	C.T	AA.	...	..C	C.T	...	...
Tupaia tana	...	...	...	TA.	...	...	...	...	...	...	GT.	...	...	C.T	AA.	...	..C	C.T	...	...
Tupaia longipes	...	...	..A	TA.	...	...	...	...	...	...	GT.	...	...	C.T	AA.	...	..C	C.T	...	...
Tupaia montana	...	...	...	TA.	...	...	...	...	...	...	GT.	...	...	C.T	AA.	...	..C	C.T	...	...
Tupaia gracilis	...	...	...	TA.	...	...	...	...	...	...	GT.	...	...	C.T	AA.	...	..C	C.T	...	...

OPN1SW	**Exon 1**											**114**		**116**		**118**			**|Exon 2**	
			105					110					115					120		
Homo sapiens	TTC	GTC	TTC	GGT	CGC	CAT	GTT	TGT	GCT	TTG	GAG	**GGC**	TTC	**CTG**	GGC	**ACT**	GTA	GCA	GGT	CTG
Ptilocercus lowii	---	---	---	---	---	---	---	---	---	---	---	---	---	---	---	---	---	---	--.	...
Dendrogale melanura	...	T..	..T	..C	...	TT.	A.C	...	...	...	...	.C.	...	A..	...	..A	TGT	...	.--	---
Dendrogale murina	...	T..	..T	..C	...	TT.	A.C	...	...	...	...	.C.	...	A..	...	..A	TGT	...	...	---
Tupaia everetti	...	T..	..T	..C	...	TT.	A..	...	.GC	C..	..A	.CT	...	A..	...	..C	...	A..	.--	---
Tupaia minor	...	T..	..T	..C	...	TT.	A..	...	.GC	C..	..A	.CT	...	A..	...	..C	...	...	...	---
Tupaia belangeri	...	T..	..T	..C	...	TT.	A..	...	.G.	C..	..A	.CT	...	A..	...	...	...	...	...	...
Tupaia tana	...	T..	..T	..C	...	TT.	A..	...	.GC	C..	..A	.CT	...	A..	...	..C	...	...	...	...
Tupaia longipes	...	T..	..T	..C	...	TT.	A..	...	.G.	C..	..A	.C.	G..	A..	...	..C	...	...	...	...
Tupaia montana	...	T..	..T	..C	...	TT.	A..	...	.G.	C..	..A	.CT	...	A..	...	..C	...	...	...	...
Tupaia gracilis	...	T..	..T	..C	...	TT.	A..	...	.G.	C..	..A	.CT	...	A..	...	..C	...	...	...	---

OPN1SW	**Exon 2**																			
			125					130					135					140		
Homo sapiens	GTT	ACA	GGA	TGG	TCA	CTG	GCC	TTC	CTG	GCC	TTT	GAG	CGC	TAC	ATT	GTC	ATC	TGT	AAG	CCC
Ptilocercus lowii	..G	.T.	..C	...	.A.	.C.	...	...	...	.A-	---	.G.	...	...	.C.	...	...	...	...	...
Dendrogale melanura	---	---	---	---	---	---	---	---	---	---	---	---	---	---	---	---	---	---	---	---
Dendrogale murina	---	---	---	---	...	T..	..A	...	...	...	...	..A	...	...	..C	...	...	...	...	...
Tupaia everetti	---	---	---	-..	...	T..	..A	...	...	...	...	...	...	...	...	...	...	...	...	...
Tupaia minor	---	---	---	---	---	---	---	---	---	---	---	---	---	---	---	---	---	---	---	---
Tupaia belangeri	..G	.T.	..C	...	...	T..	..A	...	...	...	...	...	...	...	...	...	...	...	...	...
Tupaia tana	..G	.T.	..C	...	...	T..	..A	...	...	...	...	...	...	...	...	...	...	...	...	...
Tupaia longipes	..G	.T.	..C	...	...	T..	..A	...	...	...	...	...	...	...	...	...	...	...	...	...
Tupaia montana	..G	.T.	..C	...	...	T..	..A	...	...	...	...	...	...	...	...	...	...	...	...	...
Tupaia gracilis	---	---	---	---	---	---	---	---	---	---	---	---	---	---	---	---	---	---	---	---

OPN1SW **Exon 2**

			145					150					155					160		
Homo sapiens	TTC	GGC	AAC	TTC	CGC	TTC	AGC	TCC	AAG	CAT	GCA	CTG	ACG	GTG	GTC	CTG	GCT	ACC	TGG	ACC
Ptilocercus lowii	...	AA.	...	...	...	...	.A.	...	...	...	.AC	...	---	...	A..	...	A..	...	..A	...
Dendrogale melanura	---	---	---	---	---	---	---	---	---	---	---	---	---	---	---	---	---	---	---	---
Dendrogale murina	...	...	...	...	...	...	...	...	...	...	..T	...	.TC	...	...	...	...	...	...	...
Tupaia everetti	...	...	...	...	...	...	...	...	...	...	..T	...	.TC	...	...	...	A..	...	...	...
Tupaia minor	---	---	---	---	---	---	---	---	---	---	---	---	---	---	---	---	---	---	---	---
Tupaia belangeri	...	...	...	...	...	...	...	...	...	...	..T	...	.TC	...	...	...	A..	...	...	...
Tupaia tana	..T	...	...	...	...	...	...	...	...	...	..T	...	.TC	..A	...	...	A..	...	...	...
Tupaia longipes	...	...	...	...	...	...	...	...	...	...	..T	...	.TC	...	...	...	A..	...	...	...
Tupaia montana	...	...	...	...	...	...	...	...	...	...	..T	...	.TC	...	...	...	A..	...	...	...
Tupaia gracilis	---	---	---	---	---	---	---	---	---	---	---	---	---	---	---	---	---	---	---	---

OPN1SW **Exon 2** **| Exon 3**

			165					170					175					180		
Homo sapiens	ATT	GGT	ATT	GGC	GTC	TCC	ATC	CCA	CCC	TTC	TTT	GGC	TGG	AGC	CGG	TTC	ATC	CCT	GAG	GGC
Ptilocercus lowii	...	A..	...	...	A..	...	...	...	...	...	...	...	...	...	A.-	---	---	---	---	---
Dendrogale melanura	---	---	---	---	---	---	---	---	---	---	---	---	---	---	---	---	---	---	---	---
Dendrogale murina	...	...	...	...	...	...	...	...	...	...	...	...	...	...	...	...	...	..C	...	...
Tupaia everetti	...	...	...	..T	...	...	...	...	...	...	...	...	...	...	...	...	...	..C	...	...
Tupaia minor	---	---	---	---	---	---	---	---	---	---	---	---	---	---	---	...	...	..C	...	...
Tupaia belangeri	...	...	...	..T	...	...	...	...	...	...	...	...	...	...	...	...	...	...	...	...
Tupaia tana	...	...	...	..T	...	...	...	...	...	...	...	...	...	...	...	...	...	..C	...	...
Tupaia longipes	...	...	...	..T	...	...	...	...	...	...	...	...	...	...	...	...	...	..C	...	...
Tupaia montana	...	...	...	..T	...	...	...	...	...	...	...	...	...	...	..-	---	---	---	---	---
Tupaia gracilis	---	---	---	---	---	---	---	---	---	---	---	---	---	---	---	t..	...	..C	...	...

OPN1SW **Exon 3**

			185					190					195					200		
Homo sapiens	CTG	CAG	TGT	TCC	TGT	GGC	CCT	GAC	TGG	TAC	ACC	GTG	GGC	ACC	AAA	TAC	CGC	AGC	GAG	TCC
Ptilocercus lowii	---	---	---	---	---	---	---	---	---	---	---	---	---	---	---	---	---	---	---	---
Dendrogale melanura	---	---	---	---	---	---	---	---	---	---	---	---	---	---	---	---	---	---	---	---
Dendrogale murina	...	...	..C	...	..C	...	..C	...	...	...	...	...	...	...	..G	...	...	...	...	.A.
Tupaia everetti	...	...	...	...	...	...	..C	...	...	...	...	...	...	...	...	...	...	...	...	.A.
Tupaia minor	...	...	...	...	...	...	..C	...	...	...	...	...	...	...	...	...	...	...	...	.A.
Tupaia belangeri	...	...	...	...	...	...	..C	...	...	...	...	...	...	...	...	...	...	...	...	.A.
Tupaia tana	...	...	...	...	...	...	..C	...	...	...	...	...	...	...	...	...	...	...	...	.A.
Tupaia longipes	...	...	...	...	...	...	..C	...	...	...	...	...	...	...	...	...	...	...	...	.A.
Tupaia montana	---	---	---	---	---	---	---	---	---	---	---	---	---	---	---	---	---	---	---	---
Tupaia gracilis	...	...	...	...	...	...	..C	...	...	...	..T	...	...	...	...	...	...	...	...	.A.

OPN1SW **Exon 3**

			205					210					215					220		
Homo sapiens	TAT	ACG	TGG	TTC	CTC	TTC	ATC	TTC	TGC	TTC	ATT	GTG	CCT	CTC	TCC	CTC	ATC	TGC	TTC	TCC
Ptilocercus lowii	---	---	---	---	---	---	---	---	---	---	---	---	---	---	---	---	---	---	---	---
Dendrogale melanura	---	---	---	---	---	---	---	---	---	---	---	---	---	---	---	---	---	---	---	---
Dendrogale murina	..C	..C	...	...	...	...	...	...	...	...	..C	...	..G	...	G..	...	...	...	...	...
Tupaia everetti	..C	..C	...	...	...	...	...	...	...	...	...	...	...	..T	G..	...	...	...	...	...
Tupaia minor	...	..C	...	...	...	...	...	...	...	...	...	...	...	..T	G..	...	...	...	...	...
Tupaia belangeri	..C	..C	...	...	...	...	...	...	...	...	..C	...	...	..T	G..	...	...	...	...	...
Tupaia tana	..C	..C	...	...	...	...	...	...	...	...	...	...	...	..T	G..	...	...	...	...	...
Tupaia longipes	..C	..C	...	...	...	...	...	...	...	...	..C	...	...	..T	G..	...	...	...	...	...
Tupaia montana	---	---	---	---	---	---	---	---	---	---	---	---	---	---	---	---	---	---	---	---
Tupaia gracilis	..C	..C	...	...	...	...	...	...	...	...	...	...	...	..T	G..	...	...	...	...	...

OPN1SW **Exon 3** | **Exon 4**

			225					230					235					240		
Homo sapiens	TAC	ACT	CAG	CTG	CTG	AGG	GCC	CTG	AAA	GCT	GTT	GCA	GCT	CAG	CAG	CAG	GAG	TCA	GCT	ACG
Ptilocercus lowii	---	---	---	---	---	---	---	---	---	---	---	---	---	---	---	---	---	---	---	---
Dendrogale melanura	---	---	---	---	---	---	---	---	---	---	---	--.	...	...	...	...	...	...	..G	..A
Dendrogale murina	...	...	.--	---	---	---	---	---	---	---	---	---	-..	...	...	.W.	...	...	..G	..A
Tupaia everetti	...	T..	---	---	---	---	---	---	---	---	---	--T	...	...	...	...	...	...	..G	..T
Tupaia minor	...	T..	...	...	...	G..	...	..C	.G.	...	...	...	...	...	..K	...	...	...	..G	..T
Tupaia belangeri	...	T..	...	...	...	G..	...	..C	.G.	..C	...	...	...	...	...	...	...	...	..A	..T
Tupaia tana	...	T..	...	...	...	G..	...	..C	.G.	...	...	...	...	...	...	...	...	...	..G	..T
Tupaia longipes	...	T..	...	...	...	G..	...	..C	.G.	...	...	...	...	...	...	...	...	...	..G	..T
Tupaia montana	---	---	---	---	---	---	---	---	---	---	...	...	...	...	...	...	...	...	..G	..T
Tupaia gracilis	...	T..	...	...	...	G..	...	..C	.G.	...	...	...	...	...	...	...	...	...	..G	..T

OPN1SW **Exon 4**

			245					250					255					260		
Homo sapiens	ACC	CAG	AAG	GCT	GAA	CGG	GAG	GTG	AGC	CGC	ATG	GTG	GTT	GTG	ATG	GTA	GGA	TCC	TTC	TGT
Ptilocercus lowii	---	---	---	---	---	---	---	---	---	---	---	---	---	---	---	---	---	---	---	---
Dendrogale melanura	...	...	...	..C	..G	...	...	...	...	...	...	...	..G	...	...	..G	..C	...	..T	..C
Dendrogale murina	...	...	...	..C	..G	...	...	...	...	...	...	...	..G	...	...	..G	..C	...	..T	..C
Tupaia everetti	...	...	...	..C	..G	...	...	...	...	...	...	...	..G	...	...	..G	..C	...	..T	...
Tupaia minor	...	...	...	..C	..G	...	...	...	...	...	...	...	..G	...	...	..G	..C	...	..T	...
Tupaia belangeri	...	...	...	..C	..G	...	...	...	...	...	...	...	..G	...	...	..G	..C	...	..T	...
Tupaia tana	...	...	...	..C	...	...	...	...	...	...	...	...	..G	...	...	..G	..C	...	..T	...
Tupaia longipes	...	...	...	..C	..G	...	...	...	...	...	...	...	..G	...	...	..G	..C	...	..T	...
Tupaia montana	...	...	...	..C	..G	...	...	...	...	...	...	...	..G	...	...	..G	..C	...	..T	...
Tupaia gracilis	...	...	...	..C	..G	...	...	...	...	...	...	...	..G	...	...	..G	..C	...	..T	...

OPN1SW	**Exon 4**																			
			265					270					275					280		
Homo sapiens	GTC	TGC	TAC	GTG	CCC	TAC	GCG	GCC	TTC	GCC	ATG	TAC	ATG	GTC	AAC	AAC	CGT	AAC	CAT	GGG
Ptilocercus lowii	---	---	---	---	---	---	---	---	---	---	---	---	---	---	---	---	---	---	---	---
Dendrogale melanura	..G	...	...	...	...	...	..C	...	C.G	...	...	...	...	...	...	...	..G	...	..C	..C
Dendrogale murina	..G	...	...	...	...	...	..C	..G	C.G	...	...	...	...	...	...	...	..C	...	..C	..C
Tupaia everetti	..G	...	...	...	...	...	..C	...	C.G	...	...	...	...	...	...	...	..C	...	..C	..C
Tupaia minor	..G	...	...	...	...	...	..C	...	C.G	...	...	...	...	...	...	...	..C	...	..C	..C
Tupaia belangeri	..G	...	...	...	...	...	..T	...	C.G	...	...	...	...	...	...	...	..C	...	..C	..C
Tupaia tana	..G	...	...	...	...	...	..C	...	C.G	...	...	...	...	...	...	...	..C	...	..C	..C
Tupaia longipes	..G	...	...	...	...	...	..C	...	C.G	...	...	...	...	...	...	...	..C	...	..C	..C
Tupaia montana	..G	...	...	...	...	...	..T	...	C.G	...	...	...	...	...	...	...	..C	...	..C	..C
Tupaia gracilis	..G	...	...	...	...	...	..C	...	C.G	...	...	...	...	...	...	...	..C	...	..C	..C

OPN1SW	**Exon 4**																			
			285					290					295					300		
Homo sapiens	CTG	GAC	TTA	CGG	CTT	GTC	ACC	ATT	CCT	TCA	TTC	TTC	TCC	AAG	AGT	GCT	TGC	ATC	TAC	AAT
Ptilocercus lowii	---	---	---	---	---	---	---	---	---	---	---	---	---	---	---	---	---	---	---	---
Dendrogale melanura	A.A	...	C.G	A..	..C	...	...	..C	...	G.C	...	...	...	...	..C	..G	..-	---	---	---
Dendrogale murina	A.A	...	C.G	...	..G	...	...	..C	..C	G.C	...	...	...	...	..C	..G	...	G..	...	...
Tupaia everetti	A.A	...	...	...	..C	...	...	..C	...	G.C	...	...	...	...	..C	..G	...	G..	...	...
Tupaia minor	A.A	...	...	...	..C	...	...	..C	..C	G.C	...	...	...	...	..C	..G	...	G..	...	...
Tupaia belangeri	A.A	...	...	...	..C	...	...	..C	..C	G.C	...	...	...	...	..C	..G	...	G..	...	...
Tupaia tana	A.A	...	...	...	..C	...	...	..C	..C	G.C	...	...	...	...	..C	..G	...	G..	...	...
Tupaia longipes	A.A	...	...	...	..C	...	...	..C	..C	G.C	...	...	...	...	..C	..G	...	G..	...	...
Tupaia montana	A.A	...	...	...	..C	...	...	..C	..C	G.C	...	...	...	...	..C	..G	...	G..	...	...
Tupaia gracilis	A.A	...	...	...	..C	...	...	..C	..C	G.C	...	...	...	...	..C	..G	...	G..	...	...

OPN1SW	**Exon 4**									**| Exon 5**									
			305					310					315					320		
Homo sapiens	CCC	ATC	ATC	TAC	TGC	TTC	ATG	AAT	AAG	CAG	TTC	CAA	GCT	TGC	ATC	ATG	AAG	ATG	GTG	TGT
Ptilocercus lowii	---	---	---	---	---	---	---	---	---	---	---	---	---	---	---	---	---	---	---	---
Dendrogale melanura	---	---	---	---	---	---	---	---	---	---	...	.G.	..C	...	...	...	G..	...	...	.T.
Dendrogale murina	...	...	...	...	...	...	...	..A	---	...	...	.G.	..C	...	...	...	G..	...	...	...
Tupaia everetti	...	...	...	...	...	...	...	..-	---	---	...	.G.	...	...	...	...	G..	...	...	...
Tupaia minor	...	...	...	...	..M	...	...	..A	.--	---	...	.G.	...	...	...	...	G..	...	...	...
Tupaia belangeri	...	...	...	...	...	...	...	...	...	...	...	.G.	...	...	...	...	G..	...	...	...
Tupaia tana	...	...	...	...	...	...	...	...	...	...	...	.G.	...	...	...	...	G..	...	...	...
Tupaia longipes	...	...	...	...	...	...	...	...	...	...	...	.G.	...	...	...	...	G..	...	...	...
Tupaia montana	...	...	...	...	...	...	...	...	...	...	...	.G.	...	...	...	...	G..	...	...	...
Tupaia gracilis	...	...	...	...	...	...	...	...	...	...	...	.G.	...	...	...	...	G..	...	...	...

OPN1SW	**Exon 5**																			
			325					330					335					340		
Homo sapiens	GGG	AAG	GCC	ATG	ACA	GAT	GAA	TCC	GAC	ACA	TGC	AGC	TCC	CAG	AAA	ACA	GAA	GTT	TCT	ACT
Ptilocercus lowii	---	---	---	---	---	---	---	---	---	---	---	---	---	---	---	---	---	---	---	---
Dendrogale melanura	A..	...	C..	...	...	..C	...	..G	..A	.TG	.C.	...	..T	...	.G.	..C	...	..C	...	..C
Dendrogale murina	A..	...	C..	...	...	..C	...	..G	..A	.TG	.C.	...	..T	...	.G.	...	...	..C	...	..C
Tupaia everetti	AA.	...	C..	...	...	..C	...	..T	..A	.TG	.C.	...	..T	...	.G.	..G	...	...	...	..C
Tupaia minor	A..	...	C..	...	...	..C	...	..T	..A	.TG	.C.	...	..T	...	.G.	...	...	...	...	..C
Tupaia belangeri	A..	...	C..	...	...	..C	...	..T	..A	.TG	.C.	...	..T	...	.G.	...	...	...	...	..C
Tupaia tana	A..	...	C..	...	...	..C	...	..T	..A	.TG	.C.	...	..T	...	.G.	..G	...	...	...	...
Tupaia longipes	A..	...	C..	...	...	..C	...	..T	..A	.TG	.C.	...	..T	...	.G.	..G	...	...	...	..Y
Tupaia montana	A..	...	C..	...	...	..C	...	..T	..G	.TG	.C.	...	..T	...	.G.	..G	...	...	...	..C
Tupaia gracilis	A..	...	C..	...	...	..C	...	..T	..G	.TG	.CT	...	..T	...	.G.	..G	...	...	...	..C

```
OPN1SW                 Exon 5
                                 345                                 350
Homo sapiens           GTC TCG TCT ACC CAA GTT GGC CCC AAC TGA
Ptilocercus lowii      --- --- --- --- --- --- --- --- --- ---
Dendrogale melanura    ... ..T ... .G. ... ... ... ... ... .A.
Dendrogale murina      ... ..T ... .G. ... ... ... ... ... .A.
Tupaia everetti        ... ..T ..C .G. ... ... ... ... ... .A.
Tupaia minor           ... ..T ..C .G. ... ... ... ... ... .A.
Tupaia belangeri       ... ..T ..C .G. ... ... ... ... ... .A.
Tupaia tana            ... ..T ..C .G. ... ... ... ... ... .A.
Tupaia longipes        ... ..T ..C .G. ... ... ... ... ... .A.
Tupaia montana         ... ..T ..C .G. ... ... ... ... ... .A.
Tupaia gracilis        ... ..T ..C .G. ... ... ... ... ... .A.
```

Amino acid sequences

```
OPN1SW                 Exon 1                                                      46 49 52
                                10         20         30         40         50         60         70
Homo sapiens           MRKMSEEE-F YLFKNISSVG PWDGPQYHIA PVWAFYLQAA FMGTVFLIGF PLNAMVLVAT LRYKKLRQPL
Ptilocercus lowii      ---------- ---------- ---------- ---------- ---------- ---------- ----------
Dendrogale melanura    ---------- ---------- -----L..L. .....H.... ...F..FV.T ....S..... M..R......
Dendrogale murina      ---------- ---------- ------..L. .....H.... ...F..FV.T ....S..... M..R......
Tupaia everetti        ---------- ---------- -----L..L. .....H.... ...F..FV.T ....S..... M..R......
Tupaia longipes        IS......E. .....A.L.. ........L. .....H.... ...F..FV.T ....S....- M..R......
Tupaia belangeri       IS......E. .....A.L.. ........L. .....H.... ...F..FV.T ....S..... M..R......
Tupaia gracilis        IS......E. .....A.L.. ........L. .....H.... ...F..FV.T ....S..... M..R......
Tupaia minor           IS......E. .....A.L.. ........L. .....H.... ...F..FV.T ....S..... M..R......
Tupaia tana            IS......E. .....A.L.. ........L. .....H.... ...F..FV.T ....S..... M..R......
Tupaia montana         IS......E. .....A.L.. ........L. .....H.... ...F..FV.T ....S..... M..R......
```

```
OPN1SW                  Exon 1         86   90   93  97                        114 116 118|  Exon 2
                                    80         90           100          110          120          130          140
Homo sapiens            NYILVNVSFG GFLLCIFSVF PVFVASCNGY FVFGRHVCAL EGFLGTVAGL VTGWSLAFLA FERYIVICKP
Ptilocercus lowii       ---------- ---------- ---------- ---------- ---------. .I..*P...- -G..T.....
Dendrogale melanura     ......I... ...Y...... V..IN..H.. .F...FI... .A.M..C.-- ---------- ----------
Dendrogale murina       ......I... ...Y...... V..IN..H.. .F...FI... .A.M..C..- ----...... ..........
Tupaia everetti         ......I... ...Y...... V..IN..H.. .F...FI.G. .A.M...T-- ----...... ..........
Tupaia longipes         ......I... ...Y...... V..IN..H.. .F...FI.G. .AVM...... .I........ ..........
Tupaia belangeri        ......I... ...Y...... V..IN..H.. .F...FI.G. .A.M...... .I........ ..........
Tupaia gracilis         ......I... ...Y...... V..IN..H.. .F...FI.G. .A.M.....- ---------- ----------
Tupaia minor            ......I... ...Y...... V..IN..H.. .F...FI.G. .A.M.....- ---------- ----------
Tupaia tana             ......I... ...Y...... V..IN..H.. .F...FI.G. .A.M...... .I........ ..........
Tupaia montana          ......I... ...Y...... V..IN..H.. .F...FI.G. .A.M...... .I........ ..........
```

```
OPN1SW                  Exon 2                                  | Exon 3
                                150          160          170          180          190          100          210
Homo sapiens            FGNFRFSSKH ALTVVLATWT IGIGVSIPPF FGWSRFIPEG LQCSCGPDWY TVGTKYRSES YTWFLFIFCF
Ptilocercus lowii       .N....N... D.-.I.T.*. .S..I..... ....------ ---------- ---------- ----------
Dendrogale melanura     ---------- ---------- ---------- ---------- ---------- ---------- ----------
Dendrogale murina       .......... ..I....... .......... .......... .......... .........Y ..........
Tupaia everetti         .......... ..I...T... .......... .......... .......... .........Y ..........
Tupaia longipes         .......... ..I...T... .......... .......... .......... .........Y ..........
Tupaia belangeri        .......... ..I...T... .......... .......... .......... .........Y ..........
Tupaia gracilis         ---------- ---------- ---------- -----..... .......... .........Y ..........
Tupaia minor            ---------- ---------- ---------- -----..... .......... .........Y ..........
Tupaia tana             .......... ..I...T... .......... .......... .......... .........Y ..........
Tupaia montana          .......... ..I...T... .......... ....------ ---------- ---------- ----------
```

```
OPN1SW                  Exon 3                | Exon 4
                                 220        230        240        250        260        270        280
Homo sapiens            IVPLSLICFS YTQLLRALKA VAAQQQESAT TQKAEREVSR MVVVMVGSFC VCYVPYAAFA MYMVNNRNHG
Ptilocercus lowii       ---------- ---------- ---------- ---------- ---------- ---------- ----------
Dendrogale melanura     ---------- ---------- --........ .......... .......... ........L. ..........
Dendrogale murina       ....A..... ..-------- ---..-.... .......... .......... ........L. ..........
Tupaia everetti         ....A..... .S-------- --........ .......... .......... ........L. ..........
Tupaia longipes         ....A..... .S...G..R. .......... .......... .......... ........L. ..........
Tupaia belangeri        ....A..... .S...G..R. .......... .......... .......... ........L. ..........
Tupaia gracilis         ....A..... .S...G..R. .......... .......... .......... ........L. ..........
Tupaia minor            ....A..... .S...G..R. ....-..... .......... .......... ........L. ..........
Tupaia tana             ....A..... .S...G..R. .......... .......... .......... ........L. ..........
Tupaia montana          ---------- ---------- .......... .......... .......... ........L. ..........
```

```
OPN1SW                  Exon 4                           | Exon 5
                                 290        300        310        320        330        340        350
Homo sapiens            LDLRLVTIPS FFSKSACIYN PIIYCFMNKQ FQACIMKMVC GKAMTDESDT CSSQKTEVST VSSTQVGPN*
Ptilocercus lowii       ---------- ---------- ---------- ---------- ---------- ---------- ----------
Dendrogale melanura     I........A ......---- ---------- .R....E..F R.P.....EM S...R..... ...S......
Dendrogale murina       I........A .......V.. ........K-. .R....E... R.P.....EM S...R..... ...S......
Tupaia everetti         I........A .......V.. .......--- .R....E... K.P.....EM S...R..... ...S......
Tupaia longipes         I........A .......V.. .......... .R....E... R.P.....EM S...R....- ...S......
Tupaia belangeri        I........A .......V.. .......... .R....E... R.P.....EM S...R..... ...S......
Tupaia gracilis         I........A .......V.. .......... .R....E... R.P.....EM S...R..... ...S......
Tupaia minor            I........A .......V.. ....-..K-- .R....E... R.P.....EM S...R..... ...S......
Tupaia tana             I........A .......V.. .......... .R....E... R.P.....EM S...R..... ...S......
Tupaia montana          I........A .......V.. .......... .R....E... R.P.....EM S...R..... ...S......
```

b) *OPN1LW*

Nucleotide sequences

OPN1LW	**Exon 3**																			
				140					145					150					155	
Homo sapiens	-GG	ATC	ACA	GGT	CTC	TGG	TCT	CTG	GCC	ATC	ATT	TCC	TGG	GAG	AGA	TGG	ATG	GTG	GTC	TGC
Ptilocercus lowii	-TC	...	..G	..G	...	...	..G	...	...	...	...	...	...	...	..G	...	C..	...	...	...
Dendrogale melanura	---	---	---	---	---	---	---	---	---	---	---	---	---	---	---	---	C..	...	...	...
Dendrogale murina	-TC	...	..G	..G	...	...	..C	...	...	...	...	...	...	...	..G	...	C..	...	...	...
Tupaia everetti	---	...	..G	..G	...	...	..C	...	...	...	...	..A	...	...	..G	...	C..	...	...	...
Tupaia longipes	-.A	...	...	..G	...	...	..C	...	...	...	...	..A	...	...	..G	...	C..	...	...	...
Tupaia belangeri	-.C	...	..G	..G	...	...	..C	...	...	...	...	..A	...	...	..G	...	C..	...	...	...
Tupaia gracilis	-..	...	...	..G	...	...	..C	...	...	...	...	..A	...	...	..G	...	C..	...	...	...
Tupaia minor	-..	...	...	..G	...	...	..C	...	...	...	...	..A	...	...	..G	...	C..	...	...	...
Tupaia tana	-.A	...	...	..G	...	...	..C	...	...	...	...	..A	...	...	..G	...	C..	...	...	...
Tupaia montana	-.A	...	...	..G	...	...	..C	...	...	...	...	..A	...	...	..G	...	C..	...	...	...

OPN1LW	**Exon 3**																			
				160					165					170					175	
Homo sapiens	AAG	CCC	TTT	GGC	AAT	GTG	AGA	TTT	GAT	GCC	AAG	CTG	GCC	ATC	GTG	GGC	ATT	GCC	TTC	TCC
Ptilocercus lowii	...	..T	...	...	...	...	...	..C	..C	...	...	...	...	..T	A..	...	..C	...	...	...
Dendrogale melanura	...	...	...	...	..C	...	...	N..	...	...	...	...	...	...	.CA	...	...	...	...	...
Dendrogale murina	...	...	...	...	..C	...	...	...	...	...	...	...	...	...	.CA	...	...	...	...	...
Tupaia everetti	...	...	...	...	..C	...	...	...	...	...	...	...	...	...	.CA	...	...	...	..T	...
Tupaia longipes	...	...	...	...	..C	...	...	...	...	...	...	...	...	...	.CA	...	...	...	..T	...
Tupaia belangeri	...	...	..C	...	..C	...	...	...	...	...	...	...	...	..T	.C.	...	...	...	..T	...
Tupaia gracilis	...	...	...	...	..C	...	...	...	...	...	...	...	...	...	.CA	...	...	...	..T	...
Tupaia minor	...	...	...	...	..C	...	...	...	..C	...	...	...	...	...	.CA	...	...	...	..T	...
Tupaia tana	...	...	...	...	..C	...	...	...	...	...	...	...	...	...	.CA	...	...	...	..T	...
Tupaia montana	...	...	...	...	..C	...	...	...	...	...	...	...	...	...	.CA	...	...	...	..T	...

OPN1LW	**Exon 3**			180												
				180					185					190		
Homo sapiens	TGG	ATC	TGG	**GCT**	GCT	GTG	TGG	ACA	GCC	CCG	CCC	ATC	TTT	GGT	TGG	AGC
Ptilocercus lowii	...	G..	...	...	...	...	...	..T	...	..A	...	...	...	...	...	...
Dendrogale melanura	...	...	...	T..	...	A.A	...	...	...	..A	...	...	...	...	...	...
Dendrogale murina	...	...	...	T..	...	A.A	...	...	...	..A	...	...	...	...	...	...
Tupaia everetti	...	...	...	...	...	...	...	...	...	..A	...	...	...	...	...	...
Tupaia longipes	...	...	...	...	...	...	...	...	...	..A	...	...	...	...	...	...
Tupaia belangeri	...	...	...	...	...	...	...	...	...	..A	...	...	...	...	...	...
Tupaia gracilis	...	...	...	...	...	...	...	...	...	..A	...	...	...	...	...	...
Tupaia minor	...	...	...	...	...	...	...	...	...	..A	...	...	...	...	...	...
Tupaia tana	...	...	...	...	...	...	...	...	...	..A	...	...	...	...	...	...
Tupaia montana	...	...	...	...	...	...	...	...	...	..A	...	...	...	...	...	...

OPN1LW	**Exon 5**																			
		250					255					260					265			
Homo sapiens	GTG	GCA	AAG	CAG	CAG	AAA	GAG	TCT	GAA	TCC	ACC	CAG	AAG	GCA	GAG	AAG	GAA	GTG	ACG	CGC
Ptilocercus lowii	---	---	---	---	---	---	---	---	---	---	---	---	T..	..T	...	...	..G	...	..A	...
Dendrogale melanura	...	...	...	...	..A	...	...	..C	..G	...	..T	...	...	..T	...	...	..G	...	..A	...
Dendrogale murina	---	---	---	---	---	---	---	---	---	---	---	-..	...	..T	...	...	..G	...	..A	...
Tupaia everetti	---	---	---	---	---	---	---	---	---	---	---	---	--.	..T	...	...	..G	...	..A	...
Tupaia longipes	...	...	...	...	...	...	...	...	..G	...	..T	...	...	..T	...	...	..G	...	..A	...
Tupaia belangeri	...	...	...	...	...	...	...	..C	..G	...	..T	...	...	..T	...	...	..G	...	..A	...
Tupaia gracilis	...	...	...	...	...	...	...	..C	..G	...	..T	...	...	..T	...	...	..G	...	..A	...
Tupaia minor	...	...	...	...	...	...	...	..C	..G	...	..T	...	...	..T	...	...	..G	...	..A	...
Tupaia tana	...	...	...	...	...	...	...	..C	..G	...	..T	...	...	..T	...	...	..G	...	..A	...
Tupaia montana	...	...	...	...	...	...	...	..C	..G	...	..T	...	...	..T	...	...	..G	...	..A	...

OPN1LW **Exon 5** **277** **285**

OPN1LW		270					275		277			280					285			
Homo sapiens	ATG	GTG	GTG	GTG	ATG	ATC	TTT	GCG	**TAC**	TGC	GTC	TGC	TGG	GGA	CCC	TAC	**ACC**	TTC	TTC	GCA
Ptilocercus lowii	...	...	..A	..C	...	G.G	.G.	...	...	...	...	...	...	..C	...	...	...	...	..T	...
Dendrogale melanura	...	...	..A	..C	...	G.G	.G.	...	...	...	...	...	...	..C	...	...	...	...	..T	...
Dendrogale murina	...	...	..A	..C	...	G.G	.G.	...	...	...	...	...	...	..C	...	...	...	...	..T	...
Tupaia everetti	...	...	..A	..C	...	G.G	.G.	...	...	...	...	...	...	..C	...	...	...	...	..T	...
Tupaia longipes	...	...	..A	..C	...	G.G	.G.	...	...	...	...	...	...	..C	...	...	...	...	..T	...
Tupaia belangeri	...	...	..A	..C	...	G.G	.G.	...	...	...	...	...	...	..C	...	...	...	...	..T	...
Tupaia gracilis	...	...	..A	..C	...	G.G	.G.	...	...	...	...	...	...	..C	...	...	...	...	..T	...
Tupaia minor	...	...	..A	..C	...	G.G	.G.	...	...	...	...	...	...	..C	...	...	...	...	..T	...
Tupaia tana	...	...	..A	..C	...	G.G	.G.	...	...	...	...	...	...	..C	...	...	...	...	..T	...
Tupaia montana	...	...	..A	..C	...	G.G	.G.	...	...	...	...	...	...	..C	...	...	...	...	..T	...

OPN1LW **Exon 5** **308**

OPN1LW		290					295					300					305			308
Homo sapiens	TGC	TTT	GCT	GCT	GCC	AAC	CCT	GGT	TAC	GCC	TTC	CAC	CCT	TTG	ATG	GCT	GCC	CTG	CCG	**GCC**
Ptilocercus lowii	..T	...	...	...	...	C..	...	..C	...	...	...	...	...	C..	C..	..C	...	...	..A	...
Dendrogale melanura	..T	...	...	...	...	C..	...	..C	...	...	...	...	...	C..	C..	..C	...	A..	..A	...
Dendrogale murina	..T	...	...	...	...	C..	...	..C	...	...	...	...	...	C..	C..	...	...	...	..A	...
Tupaia everetti	..T	...	...	...	...	C..	...	..C	...	...	...	...	...	C..	C..	..C	...	...	..A	...
Tupaia longipes	..T	...	...	...	...	C..	...	..C	...	...	...	...	...	C..	C..	..C	...	...	..A	...
Tupaia belangeri	..T	...	...	...	...	C..	...	..C	...	...	...	...	...	C..	C..	..C	...	...	..A	...
Tupaia gracilis	..T	...	...	...	...	C..	...	..C	...	...	...	...	...	C..	C..	..C	...	...	..A	...
Tupaia minor	..T	...	...	...	...	C..	...	..C	...	...	...	...	...	C..	C..	..C	...	...	..A	...
Tupaia tana	..T	...	...	...	...	C..	...	..C	...	...	...	...	...	C..	C..	..C	...	...	..A	...
Tupaia montana	..T	...	...	...	...	C..	...	..C	...	...	...	...	...	C..	C..	..M	...	...	..A	...

OPN1LW	**Exon 5**																			
		310					315					320					325			
Homo sapiens	TAC	TTT	GCC	AAA	AGT	GCC	ACT	ATC	TAC	AAC	CCC	GTT	ATC	TAT	GTC	TTT	ATG	AAC	CGG	CAG
Ptilocercus lowii	...	..C	...	...	...	...	..A	.--	---	---	---	---	---	---	---	---	---	---	---	---
Dendrogale melanura	...	..C	...	...	...	...	...	...	...	...	...	A.C	...	...	...	...	...	...	..T	...
Dendrogale murina	...	..C	...	...	...	...	..A	.A-	---	---	---	---	---	---	---	---	---	---	---	---
Tupaia everetti	...	..C	...	...	...	...	...	---	---	---	---	---	---	---	---	---	---	---	---	---
Tupaia longipes	...	..C	...	...	...	...	...	...	...	...	...	A.C	...	...	...	...	...	...	..T	...
Tupaia belangeri	...	..C	...	...	...	...	...	...	...	...	...	A.C	...	...	...	...	...	...	..T	...
Tupaia gracilis	...	..C	...	...	...	...	...	...	...	...	..T	A.C	...	...	...	...	...	...	..T	...
Tupaia minor	...	..C	...	...	...	...	...	...	...	...	...	A.C	...	...	...	...	...	...	..T	...
Tupaia tana	...	..C	...	...	...	...	...	...	...	...	...	A.C	...	..C	...	...	...	...	..T	...
Tupaia montana	...	..C	...	...	...	...	...	...	...	...	...	A.C	...	..C	...	...	...	...	..T	...

Amino acid sequences

OPN1LW	**Exon 3**						
					180		
	140	150	160	170	180		190
Homo sapiens	ITGLWSLAII	SWERWMVVCK	PFGNVRFDAK	LAIVGIAFSW	IW**A**	AVWTAPP	IFGWS
Ptilocercus lowii	....	.....L....	....	...M......	V..	....	.....
Dendrogale melanura	----------	-----L....	......-...	...A......	..S	.I.....	.....
Dendrogale murina	....	.....L....	....	...A......	..S	.I.....	.....
Tupaia everetti	....	.....L....	....	...A......	...	....	.....
Tupaia longipes	....	.....L....	....	...A......	...	....	.....
Tupaia belangeri	....	.....L....	....	...A......	...	....	.....
Tupaia gracilis	....	.....L....	....	...A......	...	....	.....
Tupaia minor	....	.....L....	....	...A......	...	....	.....
Tupaia tana	....	.....L....	....	...A......	...	....	.....
Tupaia montana	....	.....L....	....	...A......	...	....	.....

```
OPN1LW                    Exon 5                              277      285                        308
                           250        260        270        280        290        300        310
Homo sapiens              VAKQQKESES TQKAEKEVTR MVVVMIFAYC VCWGPYTFFA CFAAANPGYA FHPLMAALPA YFAKSATIYN
Ptilocercus lowii         ---------- ---....... .....VC... .......... .....H.... ....L..... .......---
Dendrogale melanura       .......... .......... .....VC... .......... .....H.... ....L..M.. ..........
Dendrogale murina         ---------- --........ .....VC... .......... .....H.... ....L..... .......---
Tupaia everetti           ---------- ---....... .....VC... .......... .....H.... ....L..... .......---
Tupaia longipes           .......... .......... .....VC... .......... .....H.... ....L..... ..........
Tupaia belangeri          .......... .......... .....VC... .......... .....H.... ....L..... ..........
Tupaia gracilis           .......... .......... .....VC... .......... .....H.... ....L..... ..........
Tupaia minor              .......... .......... .....VC... .......... .....H.... ....L..... ..........
Tupaia tana               .......... .......... .....VC... .......... .....H.... ....L..... ..........
Tupaia montana            .......... .......... .....VC... .......... .....H.... ....L-.... ..........
```

```
OPN1LW                    Exon 5
                           320
Homo sapiens              PVIYVFMNRQ
Ptilocercus lowii         ----------
Dendrogale melanura       .I........
Dendrogale murina         ----------
Tupaia everetti           ----------
Tupaia longipes           .I........
Tupaia belangeri          .I........
Tupaia gracilis           .I........
Tupaia minor              .I........
Tupaia tana               .I........
Tupaia montana            .I........
```
